# Prediction of Selected Biosynthetic Pathways for the Lipopolysaccharide Components in *Porphyromonas gingivalis*

**DOI:** 10.3390/pathogens10030374

**Published:** 2021-03-20

**Authors:** Wieslaw Swietnicki, Ron Caspi

**Affiliations:** 1 Department of Immunology of Infectious Diseases, L. Hirszfeld Institute of Immunology and Experimental Therapy of PAS, ul. R. Weigla 12, 53-114 Wroclaw, Poland; 2 Artificial Intelligence Center, SRI International, 333 Ravenswood Avenue, Menlo Park, CA 94025-3493, USA; ron.caspi@sri.com

**Keywords:** *Porphyromonas gingivalis*, structure prediction, LPS biosynthesis, O-type antigen

## Abstract

*Porphyromonas gingivalis* is an oral human pathogen. The bacterium destroys dental tissue and is a serious health problem worldwide. Experimental data and bioinformatic analysis revealed that the pathogen produces three types of lipopolysaccharides (LPS): normal (O-type), anionic (A-type), and capsular (K-type). The enzymes involved in the production of all three types of lipopolysaccharide have been largely identified for the first two and partially for the third type. In the current work, we use bioinformatics tools to predict biosynthetic pathways for the production of the normal (O-type) lipopolysaccharide in the W50 strain *Porphyromonas gingivalis* and compare the pathway with other putative pathways in fully sequenced and completed genomes of other pathogenic strains. Selected enzymes from the pathway have been modeled and putative structures are presented. The pathway for the A-type antigen could not be predicted at this time due to two mutually exclusive structures proposed in the literature. The pathway for K-type antigen biosynthesis could not be predicted either due to the lack of structural data for the antigen. However, pathways for the synthesis of lipid A, its core components, and the O-type antigen ligase reaction have been proposed based on a combination of experimental data and bioinformatic analyses. The predicted pathways are compared with known pathways in other systems and discussed. It is the first report in the literature showing, in detail, predicted pathways for the synthesis of selected LPS components for the model W50 strain of *P. gingivalis*.

## 1. Introduction

*Porphyromonas gingivalis* is a common human pathogen of the oral cavity. The bacterium, which is an obligate anaerobe, is responsible for the destruction of dental tissue, leading to periodontitis [1]. As the pathogen is difficult to grow under anaerobic conditions, research on the pathology and diseases caused by it is complicated. However, with the recent advances in genome sequencing and systems biology, it has been possible to elucidate a large part of its metabolic pathways [2]. The recently published corrected DNA sequence [3] of the original data [4] on *P. gingivalis* and the many sequences of different strains enabled a comparison of its genome and metabolome with those of other systems, specifically the well-studied *Escherichia coli* [5,6]. The analysis identified multiple targets for intervention and potential strategies for prophylactics [7]. The last aspect of the analysis is of importance as it is more cost-efficient to prevent periodontitis than to treat it.

Systems biology analysis of the metabolic network has identified conserved pathways used in lipopolysaccharide (LPS) synthesis by *P. gingivalis* [2]. The LPS component is a common part of many pathogens, and LPS fragments are used as adjuvants in many commercial vaccines. LPS could be a potential target of a vaccine against *P. gingivalis* [8,9] as it is important for the infection [10]. Currently, details of the metabolic pathways involved in LPS biosynthesis are not sufficiently known at the experimental level.

Two types of polysaccharides, the O-type (normal) and the anionic-type (A-type) carbohydrate, have been reported from all *P. gingivalis* strains [11,12]. A third polysaccharide (K-type, capsular) has also been identified in some strains [13] (Table 1).

Analysis of multiple *P. gingivalis* genomes showed that the pathogen secretes at least two proteases, the Arg-type gingipain and the Lys-type gingipain [14]. The proteins are similar and used by the bacterium to cleave host proteins, including bone tissue of teeth [15], leading to the destruction of periodontal tissue [16]. The gingipain protein sequences also contain adhesin motives, potentially responsible for the recognition of the host’s cell surfaces and binding to them [17]. Research by other groups identified a type IX secretion system (T9SS) responsible for gingipain secretion [18,19]. Blocking this system could be an indirect strategy for the prevention of periodontitis caused by *P. gingivalis* [20]. Following the translocation of the cargo proteins by the T9SS system, they are anchored to the cell surface via the A-type LPS [1]. The C-terminus of each cargo protein is amide-bonded to 3-acetamido-2,3-dideoxy-2-(seryl)amino-α-D-glucuronamide, which serves as a linking sugar [21], and the A-type LPS is attached by the PorZ protein [2].

The synthesis of the O-type and A-type antigens in *P. gingivalis* has not been sufficiently studied experimentally. Therefore, comparisons with similar systems in other pathogens are frequently used to identify associated operons and to assign putative roles to genes [22,23,24]. Multiple bioinformatic analyses of available genomes from *P. gingivalis* have been used to identify putative glycosyltransferases.

To simplify the analysis of the genomes, established gene names were used when there was a significant similarity to genes with a known name. For genes without established names, the genes were referred to as *pgnXXXX* (ATCC33277 strain), *pgXXXX* (W83 strain), or simply as *HMPREFYYYY_XXXX* when the assignment of genes was carried out by the HMMER search algorithm.

Based on similarities with other systems, the A-type antigen genes were identified as *gtfC* [21,25], *vimF* [25,26], *gtfE* [25], *gtfB* [27], *gtfF* [25], and *wbaP* [28]; the O-type antigen genes were identified as *wbaQ* [28], *gtfD* [25,29], *gtfE* [25], and *gtfB* [25]; and the K-type polysaccharide genes were identified as *pgn0223*/*pg0106* [28,30], none/*pg0110* [13], *pgn0232*/*pg0118* [13,25], and *pgn0233*/*pg0119* [13,25] in *P. gingivalis* strains ATCC33277 and W83, respectively. Lipid A-core genes were identified as *pgn0242* (*gtfG*)/*pg0129* [22,31] and *pgn1255* (*rfa*)/*pg1155* [32] in strains ATCC33277 and W83, respectively. The analysis also identified multiple glycosyltransferases with unknown functions [25,28,33,34,35].

To help in the prediction of biosynthetic pathways and the genes responsible for each step of LPS biosynthesis, the *P. gingivalis* W50 model genome was analyzed using the Pathway Tools software [36]. The analysis identified the genes involved in the biosynthesis of the O-type antigen and other lipid core components needed for LPS biosynthesis. Unfortunately, the K-type antigen and A-type antigen biosynthetic pathways could not be predicted—the former due to the lack of structural data and the latter due to the incompatible structural data presented by different groups. The work presented here is the first in the literature reporting a detailed assignment of genes and reactions for the *P. gingivalis* O-type antigen biosynthetic pathway and its core components. The implications are discussed for general genome-wide metabolic pathway prediction.

## 2. Results

### 2.1. Pathway/Genome Database Building

Pathway/Genome Databases (PGDBs) were created using the Pathway Tools software as part of the BioCyc portal. PGDBs are generated computationally by comparison of the genome annotation to manually curated data in the MetaCyc database. This type of database integrates the genomic data of an organism with its predicted metabolic network, including full reaction data, Enzyme Commission (EC) numbers [37], and metabolic pathways. PGDBs have been generated for the strains ATCC33277, F0185, F0566, F0568, F0569, F0570, TDC60, W4087, W50, and W83. Pathways specific for *P. gingivalis* LPS biosynthesis were curated manually using genomic data from strain W50.

### 2.2. LPS Biosynthetic Pathway Modeling

This part was performed for the *P. gingivalis* W50 strain as the most characterized in the literature in regards to the carbohydrate biosynthetic pathways. Details for the pathways in other strains were inferred from the reconstruction for the W50 strain based on putative orthologs.

#### 2.2.1. O-Type Antigen Biosynthetic Pathway

Biosynthetic pathways for O-type antigen production in bacteria can be divided into three major types, depending on the mechanism of O-type antigen assembly and the participating enzymes: Wzx/Wzy-dependent pathways, ATP-binding cassette transporters (ABC transporters)-dependent pathways, and synthase-dependent pathways, out of which the Wzx/Wzy-dependent pathways are by far the most common [3]. Analysis of the genome of the W50 strain and comparison with known biosynthetic pathways identified enzymes from the Wzx/Wzy-dependent pathways as present in the *P. gingivalis* W50 genome. Key enzymes of the ABC transporter-dependent pathways and the synthase-dependent pathways were not identified in the *P. gingivalis* W50 genome, suggesting that the O-type antigen in this organism is synthesized via a Wzx/Wzy-dependent pathway.

**Figure 1:**
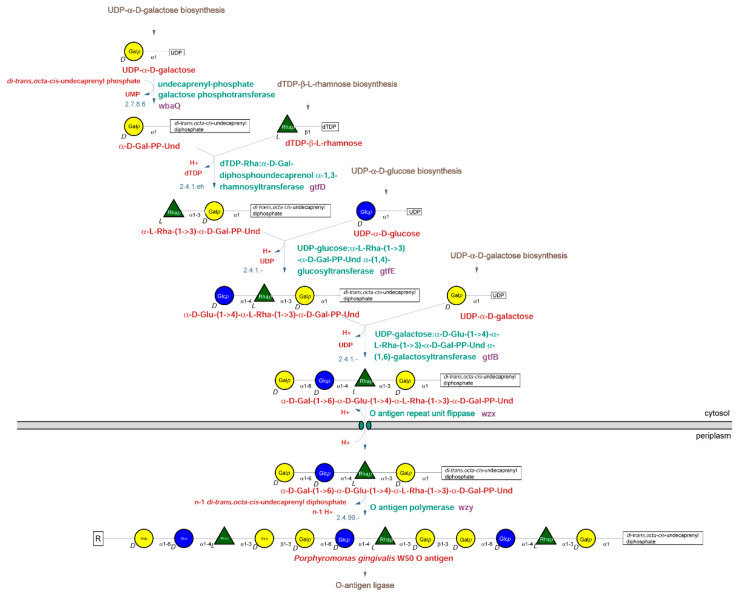
Biosynthetic pathway for O-type antigen of *P. gingivalis*. The pathway shows the synthesis of the tetrasaccharide repeat unit of the O-type antigen in the cytoplasm, its export to the periplasm, and its polymerization into the O-type antigen polysaccharide chain. Reconstruction and assignments were performed as described in the Materials and Methods section. The pathway enzymes (green) and genes (purple) are shown on the right, while EC numbers for the enzymes (blue) are shown on the left. The pathway’s intermediates are described by Glyco CT icons, with abbreviated names (red) listed underneath. Abbreviations used: Rha—rhamnose; Glc—glucose; Gal—galactose; PP-Und—di-*trans*,octa-*cis*-undecaprenyl diphosphate.

The structure of the O-type antigen repeat structure was reported in 2001 as ->3)-α-D-Gal-(1->6)-α-D-Glu-(1->4)-α -L-Rha-(1->3)-β-D-Gal-(1-> [4]. Based on the structure, the first step in the O-type antigen biosynthesis pathway of *P. gingivalis* is the transfer of a galactosyl residue from UDP-Gal*p* to di-*trans*,octa-*cis*-undecaprenylphosphate (EC 2.7.8.6) (Figure 1, top left corner).

This reaction is catalyzed in *Salmonella* species by the WbaP enzyme, which has been well characterized. *P. gingivalis* has two homologs of the *Salmonella wbaP* gene, which were named *wbaP* and *wbaQ*. Studies with different mutants suggested that WbaQ catalyzes this reaction during O-type antigen biosynthesis, while WbaP catalyzes the same reaction during A-type antigen biosynthesis [5,6].

The second predicted enzyme is dTDP-Rha:α-D-Gal-diphosphoundecaprenol α-1,3-rhamnosyltransferase. This activity is catalyzed in *Salmonella* species by the WbaN enzyme. The *P. gingivalis* enzyme was found to be encoded by the *gtfB* gene, despite little sequence similarity to *wbaN* [6].

The third residue, proposed to be glucose by Paramonov et al. [4], is transferred by the enzyme UDP-glucose:α-L-Rha-(1->3)-α-D-Gal-PP-Und α-(1,4)-glucosyltransferase. Even though Shoji et al. [6] were not certain about the identity of this residue, they identified the *gtfE* gene as encoding the transferase.

The last residue in the repeat unit proposed by Paramonov et al. [7] was galactose, which is transferred by a UDP-galactose:α-D-Glu-(1->4)-α-L-Rha-(1->3)-α-D-Gal-PP-Und α-(1,6)-galactosyltransferase. In agreement with the proposed structure, Shoji et al. proposed that the *gtfB* gene encodes a galactosyltransferase [6].

Once completed, the O-type antigen repeat unit, still attached to its lipid anchor, is flipped across the inner membrane into the periplasm by the flippase enzyme, Wzx, and multiple units are polymerized into the O-type antigen by the Wzy polymerase. The gene encoding the flippase was identified as the *HMPREF1322_RS08880* gene, and the gene encoding the polymerase was identified as the *HMPREF1322_RS01430* gene. Transferring of the polymerized antigen from its membrane anchor to the lipid A core is performed by the O antigen ligase encoded by the *HMPREF1322_RS00350* gene (*waaL*). The assignment agrees with the gene functions identified for the orthologs from the *P. gingivalis* strain ATCC33277 [5].

#### 2.2.2. Lipid A Biosynthetic Pathway

Biosynthesis of the lipid A region of LPS in *P. gingivalis* is known to produce a variety of structures. Remarkably, some of the structures act as agonists of the Toll-like receptor 2 (TLR2) and Toll-like receptor 4 (TLR4) human receptors, some are neutral, and some act as antagonists. The organism can control the distribution of the different forms in response to environmental clues such as temperature and the presence of hemin, an indicator for ulcerated tissue [14,15,16,17,18,19].

**Figure 2:**
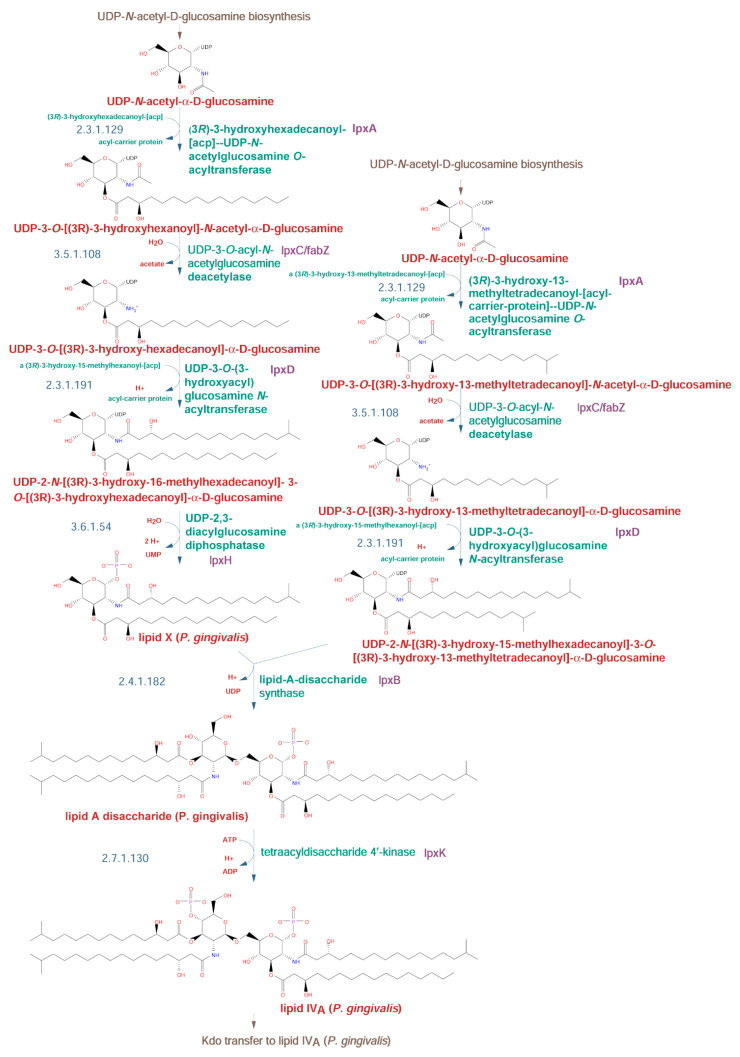
Biosynthetic pathway for lipid IV_A_ synthesis in the *P. gingivalis* W50 strain. The reactions are shown with detailed structures of substrates and products. Reconstruction and assignments were performed as described in the Materials and Methods section. The pathway enzymes (green) and genes (purple) are shown on the right, while EC numbers for the enzymes (blue) are shown on the left. Chemical compound names (red) are shown below the structures.

To simplify viewing these rather complicated biosynthetic pathways, we divided the lipid A/core biosynthesis pathway into shorter segments. The first segment is the biosynthesis of lipid IV_A_. This lipid A precursor is *N*- and *O*-acylated by four fatty acid molecules. Analysis of the W50 strain genome identified the enzymes involved in lipid IV_A_ biosynthesis (Figure 2).

The enzymes catalyzing the O- and N-acylation are LpxA (gene *HMPREF1322_RS03565*) and LpxD (gene *HMPREF1322_RS03555*), respectively [20]. Other enzymes involved are a bifunctional UDP-3-*O*-acyl-*N*-acetylglucosamine deacetylase/3-hydroxyacyl-[acyl-carrier-protein] dehydratase encoded by *lpxC* (HMPREF1322_RS03560), a diphosphatase encoded by *lpxH* (HMPREF1322_RS01980), and a kinase encoded by *lpxK* (*HMPREF1322_RS06260*).

**Figure 3:**
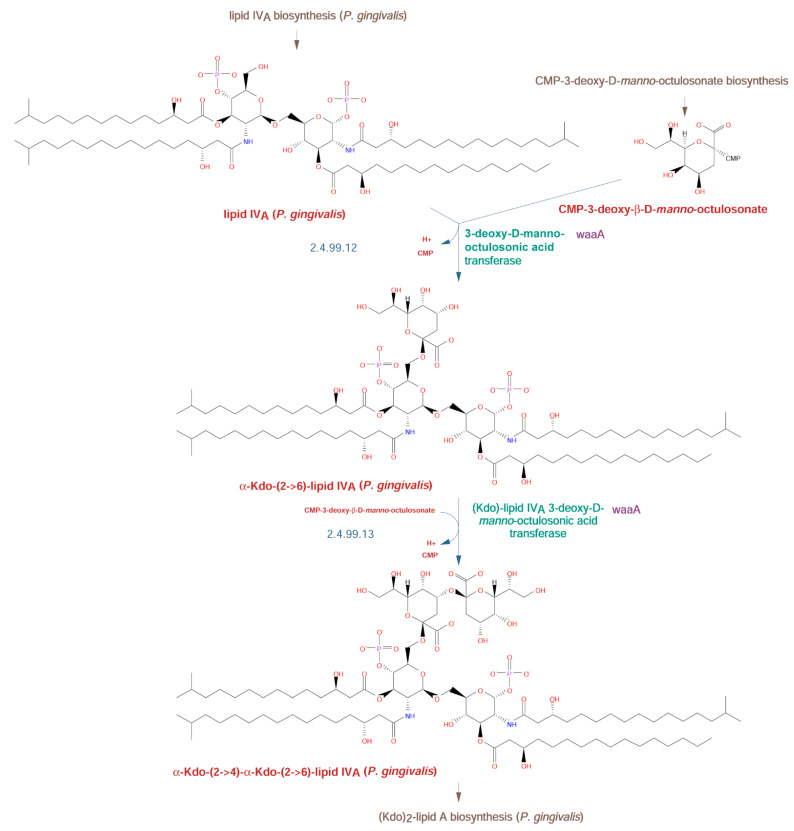
Attachment of 3-deoxy-D-manno-octulosonate (Kdo) to lipid IV_A_ in the *P. gingivalis* W50 strain. The reactions are shown with detailed structures of substrates and products. Reconstruction and assignments were performed as described in the Materials and Methods section. The pathway enzymes (green) and genes (purple) are shown on the right, while EC numbers for the enzymes (blue) are shown on the left. Chemical compound names (red) are shown below the structures.

The next step in the pathway is the attachment of 3-deoxy-D-manno-octulosonate (Kdo) residues to lipid IV_A_. Many species, such as *E. coli*, add two Kdo sugars with a single Kdo bifunctional transferase (WaaA), whereas others, including members of *Haemophilus*, *Shewanella*, and *Bacteroides vulgatus*, add a single Kdo that is then phosphorylated by a separate enzyme at its 4-OH position, the same site at which the second Kdo residue is attached in *E. coli* [8,9]. While the exact structure of the Kdo region in *P. gingivalis* lipid A has not been reported in detail, it was reported that it is not phosphorylated at position 4 [10]. Taking this into account, along with the similarity of the *waaA* gene (*HMPREF1322_RS06730*) to the *E. coli* gene, we decided to include two Kdo residues in the structure, but it is certainly possible that only a single Kdo residue is present (Figure 3).

The synthesis of (Kdo)_2_-lipid A from (Kdo)_2_-lipid IV_A_ in *P. gingivalis* is described in Figure S1. The initial reaction, catalyzed by lpxL (EC 2.3.1.241, HMPREF1322_RS07570), results in the formation of a penta-acylated, bisphosphorylated form [11]. However, the combined action of two lipid A 4′-phosphatases (lpxF, HMPREF1322_RS06645, and lptO, HMPREF1322_RS06040) [15,17], a lipid A 1-phosphatase (lpxE, HMPREF1322_RS02005) [15,38], and a lipid A deacylase (PGN_1123, HMPREF1322_RS09020) [18] results in the formation of many different forms of (Kdo)_2_-lipid A including tri-acylated (3- and 3′-deacylated) [13,22], tetra-acylated (3-deacetylated) [22,23], tetra-acylated (3′-deacetylated) [15,22,23,24], penta-acylated (bisphosphorylated) [9,21,22,24], penta-acylated (4′-dephosphorylated) [9,21,22,24], penta-acylated (1-dephosphorylated) [15], and penta-acylated (non-phosphorylated) [17,24] varieties. For simplicity, the pathway shown in Figure S1 shows the formation of only five of these forms.

The synthesis of capped core I-lipid A (penta-acylated) from (Kdo)_2_-lipid A (penta-acylated, bis-phosphorylated) is shown in Figure S2. The penta-acylated substrate is one of the versions made by the pathogen described above and used for the production of other variants. The core pathway agrees with the experimental data of Paramonov et al. [7], showing a glycerol molecule attached to a Kdo residue via a phosphoester and an unusual α-D-allosamine sugar and a tetramannose region attached to the other carbons of the glycerol core (Figure 1 in [7] and middle of Figure S2). While this structure forms the basic core region, the final structure is capped by an additional tetramannose chain that is required for the subsequent attachment of the O-type antigen. Currently, only a few of the enzymes involved in this step can be assigned to genes [7].

The final reaction of attachment of the penta-acylated capped core I-lipid A variant to the O-type antigen by the waaL ligase is shown in Figure S3. The lipopolysaccharide typically has 3–5 repeat units of the polymerized O-type antigen core and the option for the three repeat units transfer is shown.

#### 2.2.3. K-Type Antigen Biosynthetic Pathway

Analysis of the K-type antigen biosynthetic pathway in the *P. gingivalis* W50 strain is hampered by the lack of structural data for the K-type antigen, despite having the operon identified in the W83 and 381 strains [25]. Some of the steps in the W50 strain could be identified based on the predicted function of proteins, but the rest of the pathway is unknown. Putative functional assignments of proteins from the W50 operon corresponding to the genes identified in the W83 and 381 strains are given in Table 2.

The assignment of genes in the W50 strain agrees with the data published by other groups [5,6,25].

#### 2.2.4. A-Type Antigen Biosynthetic Pathway

**Figure 4:**
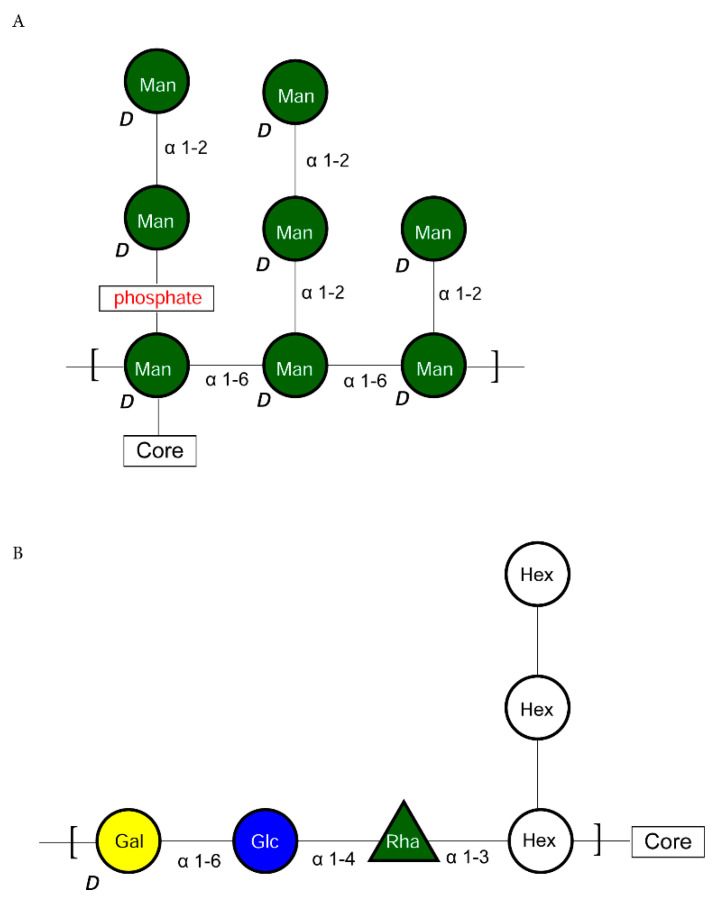
Putative A-type antigen core structures in the *P. gingivalis* W50 strain. (**A**) The structure proposed by Paramonov et al. [7]. (**B**) The structure proposed by Shoji et al. [6]. Abbreviations used: Rha—rhamnose; Glc—glucose; Gal—galactose; Hex—hexose; Man—mannose.

The biosynthesis of the A-type antigen in *P. gingivalis* has been reported by two different groups. In the work of Shoji et al. [6], the A-type antigen is based on a [unknown sugar-Rha-Glc-Gal] repeat unit (Figure 4B), with at least two enzymes, GtfE and GtfB, common for the O-type antigen synthesis, so that the last two residues added are the same as for the O-type antigen (Figure 1).

According to that study, the addition of a side branch involves at least two additional enzymes: VimF and GtfF (gene *pgn1668*). However, as the final structure of the A-type antigen was not determined by this group, the assignment of specific functions to these enzymes was not possible.

In the model of Paramonov et al. [7], the A-type antigen repeat unit consists of a backbone of three D-Man*p* units connected via α-(1->6) bonds, with three different types of side branches made of D-Man*p* units linked via α-(1->2) linkages (a single mannosyl residue, a dimannosyl side-chain connected directly to the backbone, and a dimannosyl side-chain connected via a phosphate ester). According to this model, the A-type antigen is attached to the polysaccharide core via an α-(1->4) linkage from the reducing-end mannosyl residue of the backbone to a non-terminal mannosyl residue in the core’s cap structure (Figure 4A). The evidence was based on a detailed structural analysis of a purified lipopolysaccharide by a combination of LC-MS/MS, NMR, and other techniques. Structurally, the models of the A-type antigen as reported by Shoji and Paramonov are incompatible. Reconstruction of a biosynthetic pathway for the A-type antigen production in *P. gingivalis* is, therefore, impossible at present.

### 2.3. Analysis of LPS Biosynthetic Pathways in Other P. gingivalis Strains

**Figure 5:**
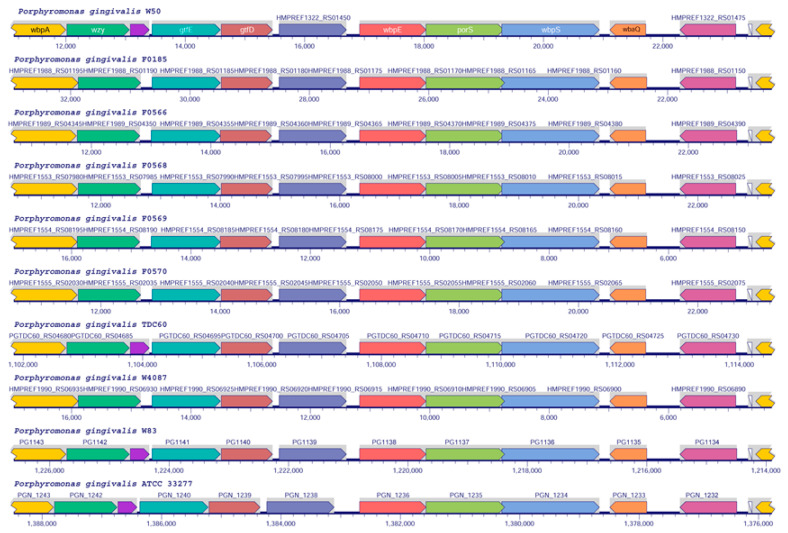
Genome region alignments of O-type antigen clusters in *P. gingivalis* strains. Clusters of putative O-type antigen biosynthetic pathways’ genes from the fully sequenced and completed genomes were aligned as described in the Materials and Methods section. The top cluster corresponds to the W50 strain with gene names according to Shoji et al. [6].

Analysis of orthologs was performed across 10 strains of *P. gingivalis* downloaded from the RefSeq database [21]. The 10 strains were W50 (NCBI Tax ID X), F0185 (NCBI Tax ID 1321821), F0566 (NCBI Tax ID 1321822), F0568 (NCBI Tax ID 1227269), F0569 (NCBI Tax ID 1227270), F0570 (NCBI Tax ID 1227271), TDC60 (NCBI Tax ID 1030843), W4087 (NCBI Tax ID 1321823), W83 (NCBI Tax ID 242619), and ATCC33277 (NCBI Tax ID 431947). The genome of strain W50 has been manually annotated for the main O-type antigen biosynthesis gene cluster, which includes the genes *wzy* (O-antigen polymerase), *gtfE* and *gtfD* (glycosyltransferases involved in O-type antigen biosynthesis), *wbpE*, *wbpS* (an aminotransferase and amidotransferase involved in the biosynthesis of 3-acetamido-2,3-dideoxy-2-(seryl)amino-α-D-glucuronamide, which serves to link A-type LPS to proteins), *porS* (a membrane protein similar to the wzx flippase with an undefined role in O-type antigen biosynthesis), and *wbaQ* (undecaprenyl-phosphate galactose phosphotransferase, the first enzyme in O-type antigen biosynthesis). Comparison of this cluster across the 10 genomes shows that it is well conserved among all organisms (Figure 5).

**Figure 6:**
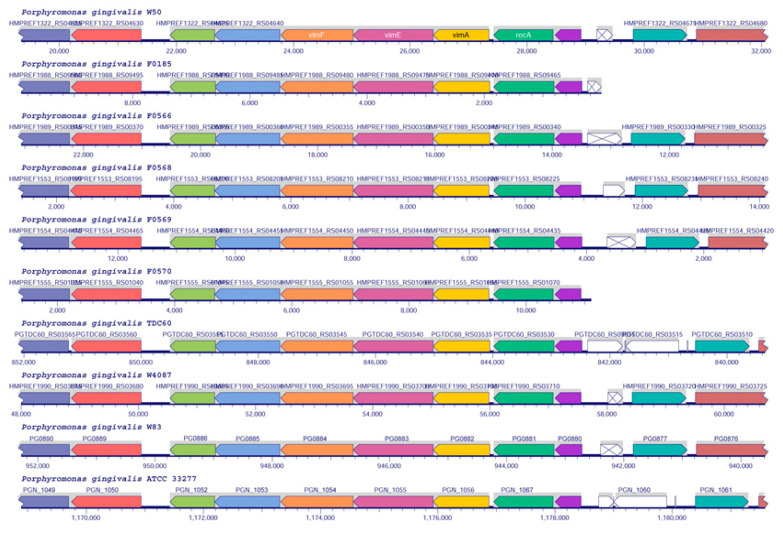
Genome region alignments of vim gene clusters in *P. gingivalis* strains. Clusters of putative O-type antigen biosynthetic pathways genes *vimA*, *vimE*, and *vimF* from the fully sequenced and completed genomes were aligned as described in the Materials and Methods section. The top cluster corresponds to the W50 strain with gene names according to Shoji et al. [6].

Another region in the genome contains the three genes *vimA*, *vimE*, and *vimF*. The *vimA* and *vimE* genes encode additional enzymes that participate in 3-acetamido-2,3-dideoxy-2-(seryl)amino-α-D-glucuronamide biosynthesis [26], while VimF catalyzes an essential but undefined activity during A-type antigen biosynthesis [27] (Figure 6).

### 2.4. Homology Modeling of Selected Components from the LPS Biosynthetic Pathway

The proteins from the LPS biosynthetic pathways have, generally, either little resemblance to the existing structures, or the similarity is too low to have a reliable homology model built. Part of the problem is the presence of transmembrane domains, either direct or based on similarities to orthologous enzymes from other bacteria. In some cases, however, the structures could be predicted and putative substrates could be docked.

#### 2.4.1. O-Antigen Biosynthetic Pathway

The first step in O-type antigen biosynthesis in *P. gingivalis*, transfer of an activated sugar residue to a phosphate group of undecaprenyl moiety, is very similar to the steps performed by the *Salmonella enterica* WbaP and the *Campylobacter jejuni* PglC enzymes [28]. The *S. enterica* enzyme N-terminal domain with four transmembrane regions is needed for its catalytic activity [29]. However, the catalytic core is located at the C-terminal domain [29,30] and has a 37% identity to the catalytic domain of *C. jejuni* PglC protein (W. Swietnicki, unpublished results). Phylogenetically, the small catalytic core is conserved in many bacterial proteins, despite differences in topologies and overall low sequence identities of proteins [31].

**Figure 7:**
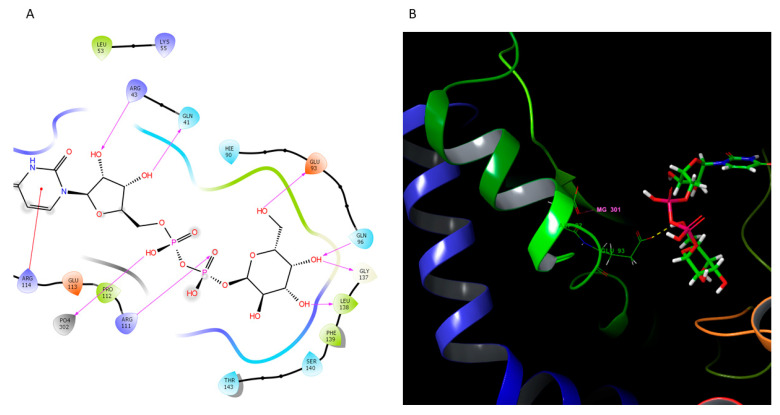
Homology modeling and putative active site docking of the WbaQ enzyme. (**A**) Active site interactions of the enzyme with a bound UDP-a-Gal*p*. (**B**) Close-up of the modeled enzyme with a bound UDP-a-Gal*p* substrate. Side-chains of the putative catalytic dyad D92-E93 are shown for orientation purposes, together with the docked Mg^+2^ ion. Part of the helix corresponding to the helix-turn-helix motive embedded in the membrane in the original PglC structure (PDB code: 5W7L) is shown on the left colored in blue.

In *P. gingivalis*, the corresponding enzyme is WbaQ and it is encoded by the *pgn1233* gene in the ATCC33277 strain [6]. The protein is responsible for the addition of galactose to the di-*trans*,octa-*cis*-undecaprenyl phosphate (Figure 1, top left corner) and has a 37% overall identity to the *C. jejuni* PglC phosphoglycosyltransferase (PDB code: 5W7L). A homology model of a monomer was constructed and the fragment a.a. 15–200 could be fitted into the existing structure (Figure 7).

The modeled protein had a very good overlap of backbone C-a atoms onto the template and the putative catalytic residues could be easily identified as D92 (Figure 7B) and E93 (Figure 7A,B). The enzyme will most likely need Mg^+2^ for catalytic activity, similar to the original PglC phosphoglycosyltransferase. The template enzyme is a membrane protein with a helix-turn-helix motive partially embedded in the membranes, the insertion needed for its catalytic activity [32]. Likely, the WbaQ enzyme will also be a membrane protein, potentially with a ping-pong (covalent intermediate) mechanism proposed for the PglC enzyme [32].

#### 2.4.2. K Antigen Biosynthetic Pathway

The gene cluster encoding K antigen biosynthesis has been reported in strain W83 [25]. Among the genes included in the cluster are genes encoding an EC 2.7.8.33, UDP-*N*-acetylglucosamine-undecaprenyl-phosphate *N*-acetylglucosaminephosphotransferase (*PG0106*), EC 5.1.3.14, UDP-*N*-acetylglucosamine 2-epimerase (non-hydrolyzing) (*PG0120*), four predicted glycosyltransferases (*PG0110*, *PG0111*, *PG0118*, and *PG0119*), two acetyltransferases (*PG0113* and *PG0115*), and a flippase (*PG0117*). Since the structure of the antigen is not known, it is not possible to assign specific functions to most of the enzymes, except for EC 2.7.8.33 and EC 5.1.3.14.

**Figure 8:**
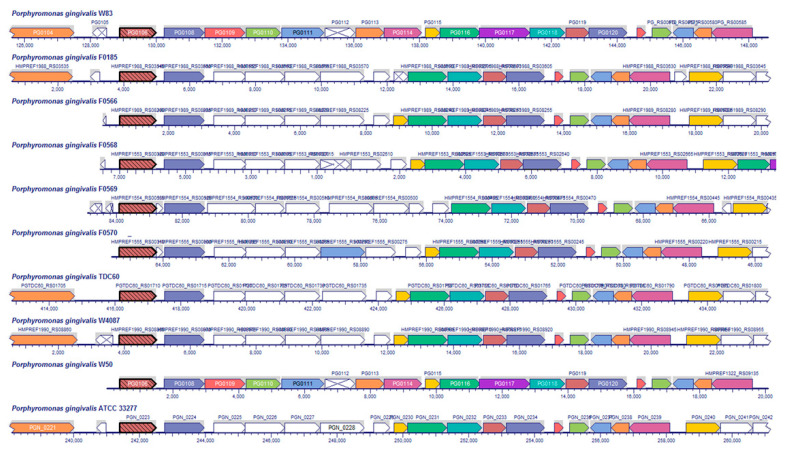
Alignment of putative K-type antigen biosynthetic pathways clusters in selected *P. gingivalis* strains. The region corresponding to the K-type antigen biosynthetic clusters as identified by Adusu-Opoke et al. [25] was aligned to corresponding regions in the fully sequenced and completed genomes of *P. gingivalis* strains as described in the Materials and Methods section.

Unlike the O-type antigen gene cluster, the K-type antigen gene cluster is not well conserved among *P. gingivalis* strains (Figure 8).

All clusters have the two starting genes (*PG0106* and *PG0108*) in addition to the four terminal genes (*PG0116–PG0120*). Based on the assigned function, only the UDP-N-acetylglucosamine-undecaprenyl-phosphate N-acetylglucosaminephosphotransferase (PG0106), flippase (*PG0117*), glycosyltransferases (*PG0118* and *PG0119*), and UDP-N-acetylglucosamine 2-epimerase (non-hydrolyzing) (*PG0120*) enzymes are conserved in all analyzed strains. The first gene (*PG0106*) may be involved in the attachment of the first sugar to the lipid anchor, but the hypothesis lacks experimental data. The presence of an additional flippase (*PG0117*) suggests that *P. gingivalis* may have a separate enzyme for flipping the K-type antigen chain across membranes in addition to the identified O-type antigen flippase *porS* (Figure 1). Based on the amino acid sequence similarities, the specificities of those enzymes are most likely different.

**Figure 9:**
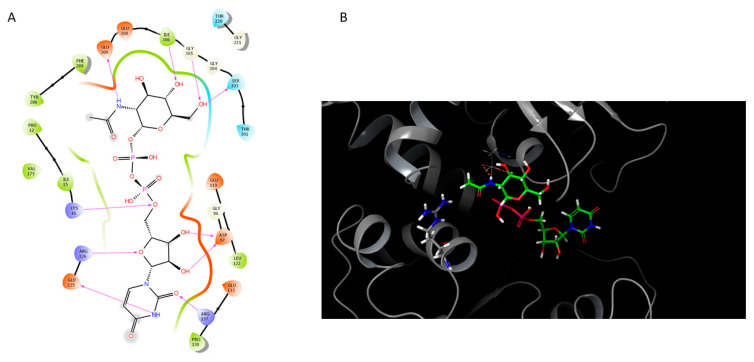
Homology modeling and putative active site docking of the UDP-N-acetylglucosamine 2-epimerase. (**A**) Active site interactions. (**B**) Close-up of the docked substrate. Residue E309 is shown behind the N-acetyl-glucosamine and putative hydrogen bonds are colored in yellow. The position of a conserved R11 residue (left, thick lines) is shown for orientation purposes.

The product of EC 5.1.3.14 is UDP-N-acetyl-D-mannosamine, an amino sugar that is found in the capsule polysaccharides of many bacterial species. The protein is encoded by the *HMPREF1322_0460* gene in the W50 strain (Table 2) and has over 60% identity to the *E. coli* UDP-N-acetylglucosamine 2-epimerase (PDB code: 1F6D). The putative model of the docked substrate, UDP-N-acetylglucosamine, in the enzyme active site is shown in Figure 9.

Analysis of the model showed that the main active site interactions (Figure 9A) agree with the structural data for the *E. coli* enzyme. Residue R11, corresponding to the residue R10 in the bacterial enzyme [33], is the closest base to the C-2 atom of the N-acetylglucosamine ring in the model and could be the catalytic residue responsible for the first step of epimerase *anti* elimination observed in bacterial enzymes [34,35]. The distance, however, is 6 Å and the domain would have to rotate, as observed in the original template (PDB code: 1F6D), for the R11 side-chain to close the distance. The model structure has the conserved G170 residue critical for the domain rotation.

## 3. Discussion

Lipopolysaccharide biosynthetic pathways have been studied for a long time in *E. coli*, and the basic pathways, together with the O-type antigen structure and enzymes involved in its synthesis, are known [39]. As DNA sequences from many organisms have become known, this information was used to predict similar pathways in other systems and to construct genomic metabolic models [40]. The models, however, lack detailed information, and since pathways vary among different organisms, using generic templates such as KEGG (https://www.genome.jp/kegg/) pathways leads to problems with reconstruction. Therefore, individual approaches that include a combination of experimental data and pathway prediction are preferred. Such a combination allows for the identification of variants and frequently explains many of the species-specific observations. In the case of *Porphyromonas gingivalis*, the information was used to elucidate lipopolysaccharide synthesis pathways and connect them to the enzymes identified by bioinformatic analysis and experimental approaches (summary in [6]). In the current work, we have identified complete pathways for the biosynthesis of O-type antigen using public information and computational analysis of whole genomes with the help of the Pathway Tools software. The combination of computational algorithms and manual curation resulted in the identification of genes, proteins, and metabolites for the model strain *Porphyromonas gingivalis* W50. The analysis was performed for the O-type antigen pathways in detail (Figure 1), including precursors used in the synthesis of core structures of LPS (Figure 2 and Figure 3). The proposed pathways are compatible with experimental data. A similar analysis for two other antigens, A-type and K-type, could not be performed due to conflicting experimental data for the former and the lack of structural data for the latter. The putative models previously proposed for the A-type antigen structure have been presented, showing their incompatibility (Figure 4A vs. Figure 4B).

A comparison with putative orthologs of other strains showed that the O-type antigen biosynthetic pathway is strictly conserved in the analyzed genomes (Figure 5). In strains W50, TDC60, W83, and ATCC33277, however, there is an extra insertion (gene *pgn1241* in the last strain). The corresponding protein does not have any similarity to the known conserved domains in the Conserved Domain Database (CDD) (https://www.ncbi.nlm.nih.gov/Structure/cdd/cdd.shtml) and its function is unknown.

A similar alignment of *vimA*, *vimF,* and *vimE* genes (Figure 6) showed that this genetic region is also strictly conserved and that there are no insertions in the analyzed strains. Since the enzymes participate in the cargo modification by the type IX secretion system or O-type antigen biosynthesis [26,27], the strict conservation may be related to the biological function of their products in the pathogenesis of *P. gingivalis*.

Analysis of putative K-type antigen biosynthetic pathways (Figure 8) showed that the possible starting point of the biosynthetic pathway (PG0106 in Figure 8) is strictly conserved in all analyzed strains, in addition to five other genes. The pathway encodes an additional flippase (PG0117, Figure 8), suggesting independence from the O-type antigen biosynthetic pathway (*wzx* in Figure 1). Based on the predicted enzyme functions, the K-type antigen may contain mannose, probably derivatized, as one of the sugar residues. The data would have to be confirmed experimentally.

Analysis of proteins for the O-type antigen biosynthetic pathway also identified a putative structure and the mechanism of reaction for the enzyme responsible for the addition of the first carbohydrate to the growing chain, the WbaQ protein. The structural information is important as the addition of other carbohydrates is most likely guided by the identity of the first residue.

This is the first report in the literature showing detailed structural information for the LPS structures being built in a human pathogen. The methodology presented here could be used to reconstruct structures for other bacterial systems and potentially speed up vaccine development significantly.

## 4. Materials and Methods

### 4.1. Genomes

The genomic data were obtained from the NCBI site for the *P. gingivalis* W50, SJD12, SJD5, W83, AJW4, JCVI SC001, SJD2, TDC60, SJD11, SJD4, F0569, F0568, F0570, F0566, 381, A7A1-28, F0185, and W4047 strains.

### 4.2. LPS Biosynthetic Pathways Reconstruction

Pathway/Genome Databases (PGDBs) were constructed for several strains of *P. gingivalis* using the Pathway Tools software [41] and included as part of the BioCyc web portal [42]. During that process, the annotated genomes, obtained from the Reference Sequence (RefSeq) database at NCBI [21], were compared to pathway and enzyme information stored in the MetaCyc database [43], and the metabolic networks of the organisms were inferred using the PathoLogic component of the Pathway Tools software [36]. The structures of the different components of the *P. gingivalis* O-type LPS (lipid A, core, O-type antigen) were reconstructed from the available data in the literature [4,17,21,22,24,43]. Pathways delineating the biosynthesis of these components were curated manually based on similar pathways characterized from other organisms and curated previously into MetaCyc. Genes were assigned to the reactions by several methods: some gene assignments were predicted by the Pathway Tools software based on genome annotation or the Pathway Hole Filler tool [44]. Other genes were assigned manually based on experimental evidence [5,6,7,11,15,17,18,38,45,46]. The remaining genes were identified by manual BLAST (https://blast.ncbi.nlm.nih.gov/Blast.cgi) searches using characterized genes from other organisms against the BioCyc *P. gingivalis* W50 database.

### 4.3. Ortholog Computation

The BioCyc project computes orthologs between two genomes using bidirectional DIAMOND comparisons across their proteomes [47]. Two proteins are inferred to be orthologs if they are the best bi-directional hits with both E-values less than 0.001. The best hit(s) of protein A in proteome PB is defined by finding the minimal E-value among all hits in proteome PB in the DIAMOND output. There could be hits to multiple proteins in proteome PB that share that same minimal E-value. In other words, ties are possible, as in the case of exact gene duplications. All ties are included in the final set of orthologs used by BioCyc. Thus, protein A could have multiple orthologs in PB, for example, if multiple proteins B1, B2, etc., exist in PB and have the same regions aligned against protein A. BioCyc does not calculate paralogs.

### 4.4. Homology Modeling of WbaQ Enzyme

The protein sequence for the *P. gingivalis* W50 strain was obtained from the NCBI site and used for homology modeling with the Modeler module within the Chimera software [48], with the PglC (PDB code: 5W7L) structure as the template. The model was optimized in Schrodinger’s Maestro suite (https://www.schrodinger.com/downloads/releases) and transferred back to Chimera for substrate docking. The structure of UDP-a-D-Gal*p* was downloaded from the PubChem website and optimized in Chimera [48] for docking, together with the WbaQ enzyme, using the AutoDock Vina module. In the first approach, the full protein surface was used for a grid search and the results were compared with the original work by Ray et al. [32]. In the second attempt, the grid search was narrowed to about 20 × 20 × 20 Å volume to match the center of the active site in the PglC structure. The best docking solution was compared with the template structure and found to agree with the proposed mechanism for the PglC enzyme. The pose was optimized, overlaid on the PglC structure, and the positions of Mg+2 and phosphate ions were used as the starting point for optimization of the WbaQ enzyme model with the docked substrate with Schrodinger’s Maestro software. The final model was displayed with the same software and the graphs were transferred to Adobe CS2 for quality adjustments. Figures were prepared with the MS Office software suite.

### 4.5. Homology Modeling of the UDP-N-Acetylglucosamine 2-Epimerase

The protein sequence was obtained from the gene *HMPREF1322_0460* (Table 2) and used for automated model building with the Swiss-Model software [49]. The final model was imported into the Chimera software [48], prepared for docking, and used for UDP-N-acetylglucosamine docking in the AutoDock Vina module. Docking poses were compared with the original structure (PDB code: 1F6D) and the first pose was selected. The docked substrate and the model were transferred into Schrodinger’s Maestro suite (https://www.schrodinger.com/downloads/releases) and adjusted in the ProteinPrep module. The final structure was examined for steric problems and no violations were found. The final model was displayed with the same software and the graphs were transferred to Adobe CS2 for quality adjustments. Figures were prepared with the MS Office software suite.

## Figures and Tables

**Table 1:** The different polysaccharides produced by the *Porphyromonas gingivalis* W50 strain.

	O-Type Antigen	A-Type Antigen	K-Type Antigen
Biological function	Protection in a hostile environment, interaction with the environment, structural support to the cell	Protection in a hostile environment	Interaction with the environment
Structural information	Fully described	Two incompatible proposals	Unknown
Genomic information	Fully described	Partially described	Locus sequenced, function of individual genes unknown

**Table 2:** Putative genes of K-type antigen biosynthetic operon in the *P. gingivalis* W50 strain.

Gene ^a^	Protein Function ^b^
*HMPREF1322_0447*	undecaprenyl/decaprenyl-phosphate α-N-acetylglucosaminyl 1-phosphate transferase
*HMPREF1322_0448*	UDP-N-acetyl-D-mannosamine dehydrogenase
*HMPREF1322_0449*	O-antigen ligase domain-containing protein
*HMPREF1322_0450*	glycosyltransferase family 1 protein
*HMPREF1322_0451*	hypothetical protein
*HMPREF1322_0454*	aminoglycoside 3-N-acetyltransferase
*HMPREF1322_0455*	hypothetical protein
*HMPREF1322_RS09085*	serine acetyltransferase
*HMPREF1322_0456*	hypothetical protein
*HMPREF1322_0457*	hypothetical protein
*HMPREF1322_0458*	glycosyltransferase family 2 protein
*HMPREF1322_0459*	glycosyltransferase
*HMPREF1322_0460*	UDP-N-acetylglucosamine 2-epimerase (non-hydrolyzing)

^a^ Gene assignment was based on the *PG0106*->*PG0120* operon correspondence between the W83 and W50 strains of *P. gingivalis*. Pseudogenes were omitted from the list. ^b^ Protein function assignment is based on the similarity to proteins with a known function as determined by the Pathway Tools software.

## Data Availability

The pathway and ortholog data useed in this manuscrpt are available at the BioCyc web portal (https://biocyc.org/organism-summary?object=GCF_000271945-HMP). Access to the data requires subscription.

## Supplementary Materials

The following are available online at https://www.mdpi.com/2076-0817/10/3/374/s1, Figure S1: The synthesis of (Kdo)_2_-lipid A in the *P. gingivalis* W50 strain; Figure S2: Synthesis of the capped core I-lipid A from the (Kdo)_2_-lipid A in the *P. gingivalis* W50 strain; Figure S3: Attachment of the capped core I-lipid A to the O-type antigen in the *P. gingivalis* W50 strain.
