# Supplementary figures and images for: Prediction of Selected Biosynthetic Pathways for the Lipopolysaccharide Components in Porphyromonas gingivalis
Source: Pathogens. 2021 Mar 20;10(3):374. doi: 10.3390/pathogens10030374 (PMC8003790; doi:10.3390/pathogens10030374)

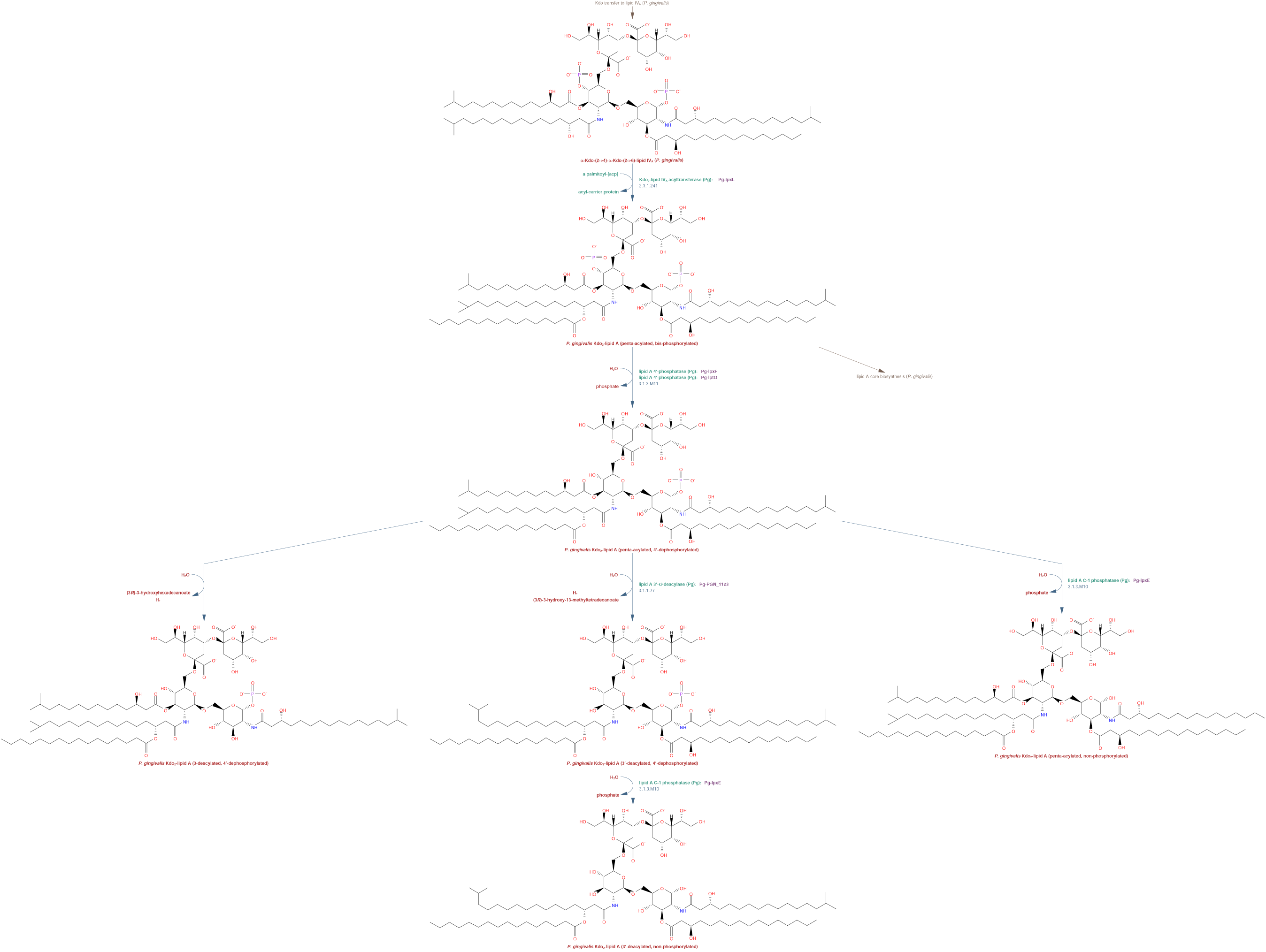

Supplement: Supplementary file 1 [file pathogens-10-00374-s001.zip › Figure S1- Kdo2-lipid A biosynthesis.tif]

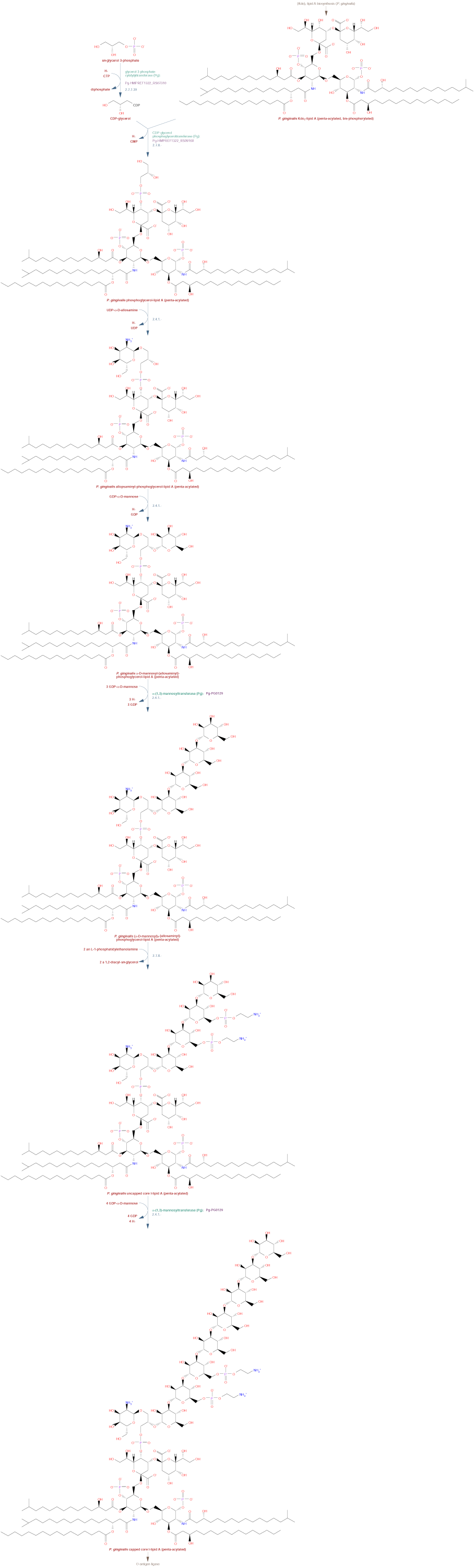

Supplement: Supplementary file 1 [file pathogens-10-00374-s001.zip › Figure S2-lipid A-core biosynthesis.tif]

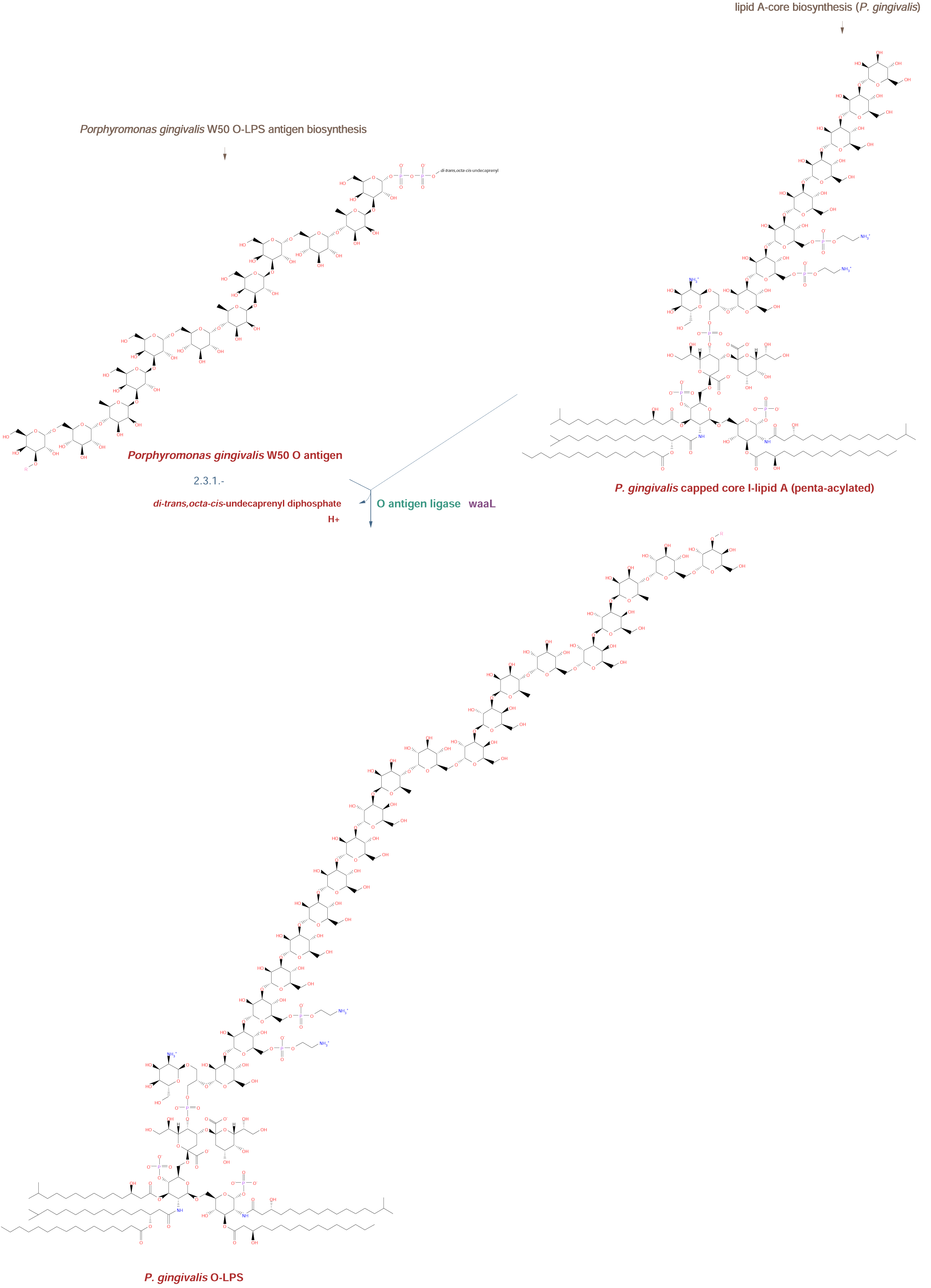

Supplement: Supplementary file 1 [file pathogens-10-00374-s001.zip › Figure S3-O-antigen ligation to lipid A-core.tif]
